# Primary Biliary Cholangitis: A Case of Underdiagnosis

**DOI:** 10.7759/cureus.17716

**Published:** 2021-09-04

**Authors:** Sofia Garcês Soares, Marina Mendes, Sofia Rodrigues Carvalho, Diana Pereira Anjos, Ana João Sá

**Affiliations:** 1 Internal Medicine, Centro Hospitalar Tâmega e Sousa, Penafiel, PRT

**Keywords:** primary biliary cholangitis, cholestasis, autoimmunity, liver disease, alkaline phosphatase, anti-mitochondrial antibodies

## Abstract

Primary biliary cholangitis is a rare autoimmune cholestatic disease with a variable clinical course. Its etiopathogenesis has not been completely clarified. It predominantly affects women and often progresses to liver cirrhosis. It may be asymptomatic or symptomatic with hepatic or extrahepatic manifestations. If its diagnosis and treatment are made early, the progression to cirrhosis and liver failure can be prevented.

We describe a clinical case of a 63-year-old woman, with no history of hepatotoxic drugs, who presented an analytical pattern of cytocholestasis with more than one decade of evolution.

## Introduction

Primary biliary cholangitis (PBC), formerly called primary biliary cirrhosis, is a rare chronic autoimmune cholestatic disease with a variable clinical course. It has a familial predisposition and it affects mainly women (~90% of the cases) between 40 and 60 years of age. Although its etiopathogenesis is not completely clear, it is thought that in genetically susceptible individuals, a viral, allergic, chemical, or pharmacological autoimmune trigger may lead to an attack of the T lymphocytes to small and medium caliber intralobular canals leading to progressive inflammation and eventually to liver cirrhosis [1-4].

It is often asymptomatic (in >60% of the cases) and it might only manifest by changes in liver tests in routine analyses [1,5]. It may have hepatic and extra-hepatic manifestations (in up to 73% of patients) [3]. The main clinical manifestations are fatigue, pruritus, jaundice, concurrent autoimmune diseases, and osteopenia/osteoporosis. The main biochemical changes are increased alkaline phosphatase (ALP), gamma-glutamyltransferase (GGT), bilirubin, and cholesterol [1,2,6].

## Case presentation

We describe a case of a 63-year-old woman, independent in the activities of daily living, with no significant past medical history, who was referred to the internal medicine consultation due to a cholestatic laboratory pattern that had been present for approximately 10 years. She did not take any medications, drank about 20 g of alcohol per day, and had no history of other hepatotoxins. She had no symptoms and had no significant changes on physical examination.

The initial laboratory study revealed normal hemoglobin (15.1 g/dL), platelets (181 10^3/uL), renal function (urea 39 mg/dL and creatinine 0.8 mg/dL), and ionogram (sodium 140 mmol/L, potassium 4.1 mmol/L, and chloride 106 mmol/L); normal sedimentation rate (8 mm) with slightly elevated C-reactive protein (15.1 mg/L) and cytocholestasis pattern (glutamic oxaloacetic transaminase [GOT] 123 IU/L, glutamic pyruvic transaminase [GPT] 121 IU/L, GGT 259 IU/L, and ALP 179 UI/L) with normal total bilirubin (0.90 mg/dL), albumin (3.7 g/dL), and coagulation (international normalized ratio [INR] 0.99). She also had high total cholesterol (211 mg/dL) and normal triglycerides (103 mg/dL).

A possible autoimmune etiology was investigated, which included normal proteinogram (proteins 6.6 g/dL, albumin 56.9%, alpha 1 5.0%, alpha 2 11.3%, beta 11.4%, and gamma 15.4%) and complement (C3 122 mg/dL and C4 44 mg/dL); normal immunoglobulin A (IgA) (226 mg/dL) and immunoglobulin G (IgG) (908 mg/dL) with increased immunoglobulin M (IgM) (254mg/dL); and positive antinuclear and extractable nuclear antigen (ENA) antibodies (9.00), anti-centromere antibodies (98.0 U/mL), and anti-mitochondrial antibodies (AMAs) (1/1280) including positive anti-mitochondrial M2 (>200 U/mL).

Other causes of liver disease were also excluded: slightly elevated ferritin (336 ng/mL), normal alpha 1 antitrypsin (156 mg/dL), copper (22.59 umol/L), and ceruloplasmin (42.1 mg/dL); and immunity to hepatitis B virus (HBV) with negative hepatitis C virus (HCV) and HIV1/2 serologies.

**Figure 1:**
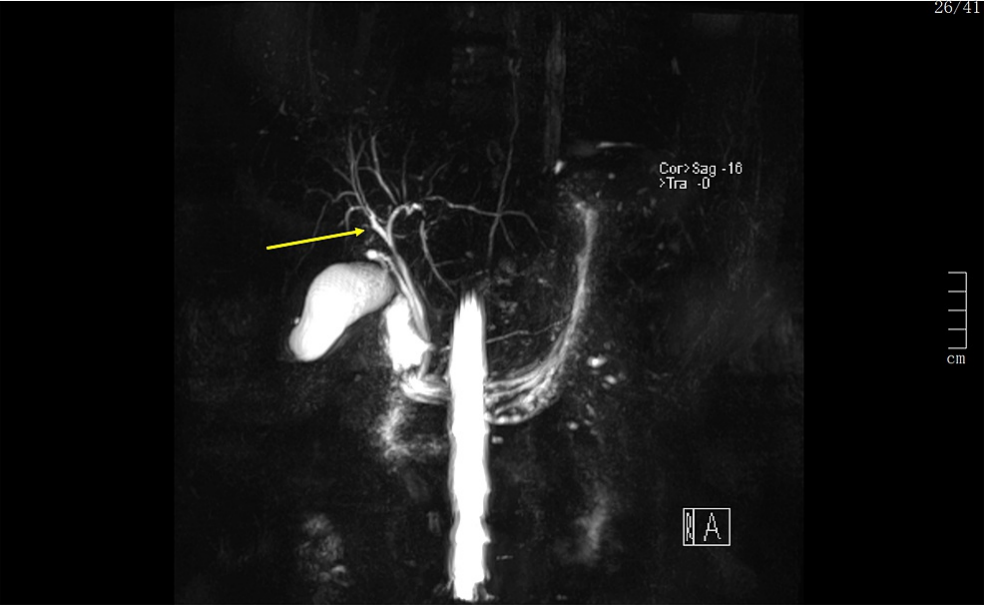
3D MRCP image showing a slight prominence of the intrahepatic bile ducts. 3D, three-dimensional; MRCP, magnetic resonance cholangiopancreatography.

An abdominal ultrasound revealed no obstructions or focal lesions, followed by a magnetic resonance cholangiopancreatography (MRCP), which revealed a slight prominence of the intrahepatic bile ducts with a decreased focal caliber of the main bile duct (Figure 1).

Given the ALP value >1.5x, the upper limit of normal and positive AMAs, primary biliary cholangitis was diagnosed.

The patient was referred for a liver transplant consultation and was started on ursodeoxycholic acid (UDCA) 250 mg bid with an improvement of the cytocholestasis laboratory pattern including ALP.

## Discussion

Although rare, PBC is a chronic disease whose prevalence has been increasing (1.91-40.20 cases/100,000 people), possibly due to greater recognition of the disease, data recording, and survival after starting treatment with UDCA [1,6].

The most common symptoms are fatigue (80% of patients) and itching (40-80% of patients). It can progress slowly and progressively over several decades and its clinical manifestations may remain stable for several years [1,2,6]. Therefore, the diagnosis should be suspected in patients with chronic elevations of GOT, GPT, and ALP in the presence or absence of symptoms and the initial physical examination should include a search for hepatomegaly, splenomegaly, and extrahepatic signs of advanced liver disease [2].

In this case, despite the patient presented cytocholestasis with more than one decade of evolution, she remained asymptomatic and with no changes on the physical examination, which contributed to the delay in her referral to a specialized consultation.

In clinical practice, the hepatic origin of ALP is usually associated with a simultaneous elevation of serum GOT and/or conjugated bilirubin [2]. Although this patient had normal bilirubin, she had elevated GOT. It should be noted that bilirubin, albumin, and prothrombin time may be normal and may only deteriorate in advanced stages [1].

In the initial study, a possible cause of extrahepatic cholestasis, usually by ultrasound, should also be ruled out. In this clinical case, in addition to the ultrasound, an MRCP was also performed. MRCP is used for the detection of intra- and extra-hepatic bile duct stenosis and dilatations and it is essential for the diagnosis of primary and secondary sclerosing cholangitis, which, in turn, is included in the differential diagnosis of patients with cholestasis [2].

After excluding extrahepatic cholestasis, the diagnosis is based on the presence of two of the following elements: (a) biochemical cholestasis with increased ALP (≥1.5x the upper limit of normality); (b) positive AMA or disease-specific antinuclear antibodies (anti-gp210 or anti-Sp100); and (c) compatible liver biopsy [1,2].

The serological hallmark of PBC is AMA (particularly type M2), a very disease-specific antibody detected in 90-95% of PBC cases [1,2,6-8]. However, its positivity alone is not enough for the diagnosis and its title is not related to the severity of the disease, so it should not be serialized [2,6].

The presence of antinuclear antibodies is equally frequent. The increase in IgM and IgG levels, but particularly in the former (as in this clinical case), is another characteristic change [1-3,6,8].

Liver biopsy is not necessary when the other two criteria are met, as in this case. It may be convenient to do this in case of doubt, suspicion of overlap with autoimmune hepatitis (prevalence 6-10%), suspicion of non-alcoholic steatohepatitis, or absence of typical antibodies [1,2].

Liver elastography allows estimating the degree of fibrosis/disease progression [1].

In 75-95% of cases, patients have cholestasis-related dyslipidemia (in the early stage, 75% of patients have total cholesterol levels >200mg/dL), and decreased bile acid secretion can lead to decreased absorption of fat-soluble vitamins (A, D, E, and K) [1,2,4,6].

Metabolic bone disease is also a common complication and osteoporosis is seen in 20-44% of patients [3].

The prevalence of other autoimmune diseases is frequent, with Sjogren's syndrome being the most common. It is also associated with scleroderma, mixed connective tissue diseases, or CREST (calcinosis, Raynaud phenomenon, esophageal dysmotility, sclerodactyly, and telangiectasia) syndrome [1,6]. In this patient, no other autoimmune pathologies were identified.

The development of cirrhosis occurs in 25% of symptomatic patients and 5% of asymptomatic patients [6].

Symptomatic patients may have a shorter course of the disease, although in general the duration from diagnosis to transplantation or death is 15-20 years [6].

The treatment of PBC is based on the correction of liver damage and the attenuation of the symptoms and consequences of chronic cholestasis [1]. The specific first-line treatment is UDCA at a dose of 13-15 mg/kg. It appears to be more effective when started at an early stage of the disease, as it increases transplant-free survival and overall survival may be similar to the general population [1-4,8].

According to Barcelona criteria, a decrease in serum ALP value of >40% from baseline or a normal value after one year of treatment with UDCA is a marker of a good long-term prognosis. There are other proposals for biochemical response to UDCA: Paris I and II criteria, Rotterdam criteria, Toronto criteria, and Beijing criteria [1,2].

For patients who are intolerant or unresponsive to treatment with UDCA after 6-12 months, obeticholic acid (second line of treatment) should be started [1,3,5].

Serum bilirubin is the best prognostic index. Patients aged <50 years and males have a worse prognosis [1,8].

When the course of the disease cannot be stopped, the final treatment is liver transplantation, which has a survival rate of >75% at five years.

Indications for liver transplantation are similar to those for other chronic liver diseases - patients with advanced disease and portal hypertension, bilirubin levels >6mg/dL, or Model for End-Stage Liver Disease (MELD) scale ≥12 [1,2,5]. Additionally, severe and intractable pruritus is still an exceptional consideration for liver transplantation [7].

PBC may recur in 20% to 33% of transplant patients [1,2,8].

## Conclusions

This is a patient with cholestasis who remained undiagnosed and untreated for over a decade, and therefore at high risk of liver failure.

In the face of changes in liver tests and, in particular, cytocholestasis without a clear cause, as in this case, a physician cannot have a passive attitude and must exhaustively pursue a diagnosis.

The authors intend to draw attention to a rare entity whose early diagnosis and treatment can prevent the evolution of liver cirrhosis, liver failure, and death in a substantial number of patients, especially in those with rapidly progressive forms.

This is an entity that can present itself not only to clinicians in the field of gastroenterology but also to more general areas such as internal medicine or primary care; hence, the importance of disseminating and promoting knowledge about this disease.
